# Crystal structures of (*E*)-4-[1-(2-carbamo­thio­yl­hydrazinyl­idene)eth­yl]phenyl acetate and (*E*)-4-[1-(2-carbamo­thio­ylhydrazinyl­idene)eth­yl]phenyl benzoate

**DOI:** 10.1107/S2056989016018983

**Published:** 2017-01-01

**Authors:** Vijayan Viswanathan, Mani Karthik Ananth, S. Narasimhan, Devadasan Velmurugan

**Affiliations:** a Centre of Advanced Study in Crystallography and Biophysics, University of Madras, Guindy Campus, Chennai 600 025, India; b Department of Chemistry, Asthagiri Herbal Research Foundation, Perungudi Industrial Estate, Perungudi, Chennai 600 096, India

**Keywords:** crystal structure, carbamo­thio­ylhydrazono, thio­semicarbazone, N—H⋯S and N—H⋯O hydrogen bonds, hydrogen bonding, N—H⋯π and C—H⋯π inter­actions

## Abstract

In the title compounds, C_11_H_13_N_3_O_2_S, (I), and C_16_H_15_N_3_O_2_S, (II), the thio­semicarbazone group adopts an extended conformation. The acetate ester (I) crystallizes with two independent mol­ecules in the asymmetric unit. In the benzoate ester (II), the planes of the two aryl rings are inclined to one another by 46.70 (7)°. In both compounds, there is a short intra­molecular N—H⋯N contact present, forming an *S*(5) ring motif. In the crystals of both compounds, mol­ecules are linked *via* pairs of N—H⋯S hydrogen bonds, forming dimers with *R*_2_^2^(8) ring motifs. The dimers are linked by N—H⋯S and N—H⋯O hydrogen bonds, forming slabs parallel to (01-1). In (I), there are N—H⋯π and C—H⋯π inter­actions present within the slabs, while in (II), there are only N—H⋯π inter­actions present.

## Abstract 2

In the title compounds, the thio­semicarbazone group adopts an extended conformation, and there is a short N—H⋯N contact present forming an *S*(5) ring motif. In the crystals of both compounds, mol­ecules are linked by pairs of N—H⋯S hydrogen bonds, forming dimers with 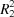
(8) ring motifs.

## Chemical context   

Thio­semicarbazones are potent inter­mediates for the synthesis of pharmaceutical and bioactive materials and they are used extensively in the field of medicinal chemistry. The biological activity of these ligands is related to their ability to coordinate to metal centres in enzymes (Seena *et al.*, 2006 ▸). These derivatives possess an additional functional group that is not coordinated to their ‘primary’ metal ion, thereby suggesting that the biological activity may also depend on the non-coordinating groups (Venkatesh *et al.*, 2016 ▸). Thio­semicarbazones in their neutral or deprotonated form behave as *N,N,S*-thio­dentate chelates towards metal ions. They display anti­proliferative activity on different tumors cell lines and have been a common feature of all compounds with carcinogenic potency. A strong correlation has been found between tumor growth rate and the ribonucleoside diphos­phate reductase (RDR) enzyme (Arora *et al.*, 2014 ▸).

Thio­semicarbazone derivatives have found applications in drug development for the treatment of central nervous system disorders and bacterial infection as well as being analgesic and anti-allergic agents. They are inhibitors of DNA replication and also of many proteases. This inhibitory activity explains the level of inter­est given to them in the fight against microbial and parasitic diseases (Mani *et al.*, 2015 ▸). Thio­semicarbazones have many biological activities such as anti­viral, anti­bacterial, anti­tumor, anti African trypanosome (Fatondji *et al.*, 2013 ▸), anti­microbial, sodium channel blocker, anti­cancer, anti­tubercular, anti­viral (Venkatesh *et al.*, 2016 ▸), anti­fungal, locomotor activity (Singh *et al.*, 2011 ▸), anti­malarial, anti­cancer and they are used as a cure for leprosy, rheumatism and trypanosomiasis (Parul *et al.*, 2012 ▸). As part of our studies in this area, we now describe the syntheses and structures of the title compounds (I[Chem scheme1]) and (II[Chem scheme1]).
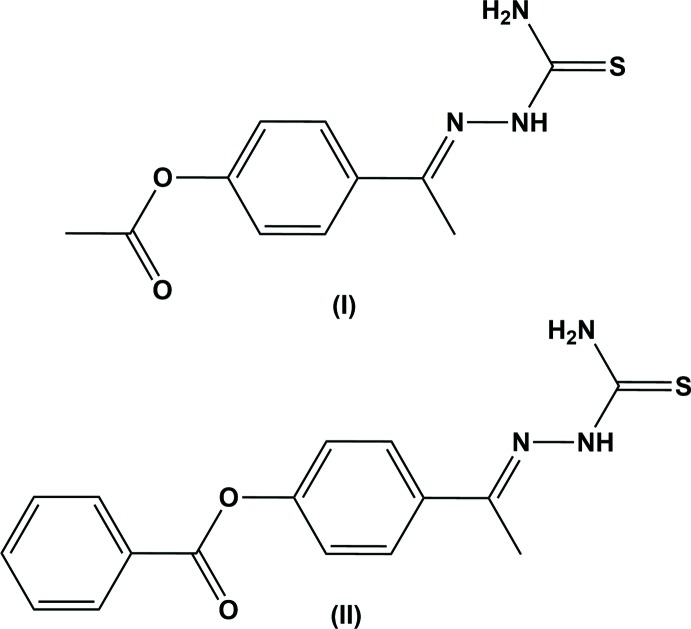


## Structural commentary   

**Figure 1:**
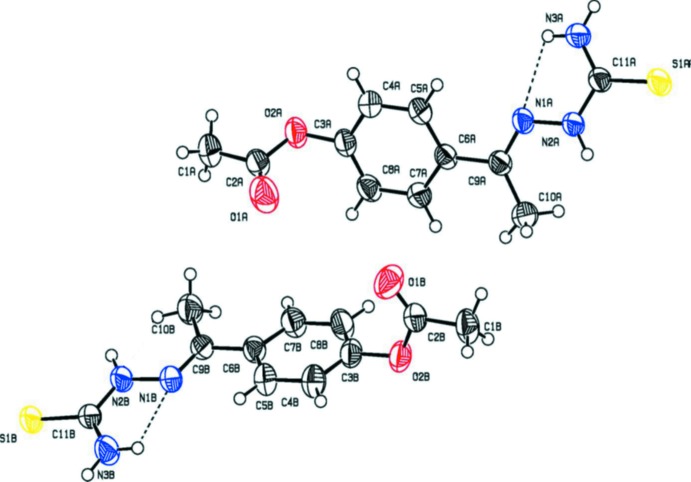
The mol­ecular structure of the compound (I), showing the atom labelling and displacement ellipsoids drawn at the 30% probability level. The short intra­molecular N—H⋯N contact is shown as a dashed line (see Table 1 ▸).

The mol­ecular structure of compounds (I) and (II) are shown in Figs. 1 ▸ and 2 ▸, respectively. Compound (I) crystallizes with two independent mol­ecules in the asymmetric unit. In both the compounds, there is a short N—H⋯N contact, forming an *S*(5) ring motif (Figs. 1 ▸ and 2 ▸, and Tables 1 ▸ and 2 ▸). In both compounds, the thio­semicarbazone group adopts an extended conformation, as can be seen from the torsion angle S1—C11—N2—N1 [−173.1 (1)° in mol­ecule *A* and −174.9 (1)° in mol­ecule *B* of compound (I)] and S1—C16—N2—N1 [172.2 (1)° in compound (II)]. In compound (I), the acetate group adopts an extended conformation, which is evidenced by the torsion angle C1—C2—O2—C3 [−173.2 (2) and 179.9 (2)° in mol­ecules *A* and *B*, respectively]. The bond lengths C11*A*—S1*A* [1.692 (2) Å] and C11*B*—S1*B* [1.680 (2) Å] in (I) and C16—S1 [1.679 (1) Å] in (II) are comparable with the values reported in the literature (CSD; Groom *et al.*, 2016 ▸). In compound (II), the benzoate and aceto­phenone thio­semicarbozone groups lie in a plane [C6—C7—O2—C8 = 175.9 (1)°]. The carbonyl group is oriented *syn*-periplanar to C5 [C5—C6—C7—O1 = −15.8 (2) °] and *anti*-periplanar to C1 [C1—C6—C7—O1 = 160.7 (1) °]. The dihedral angle between the benzene rings in compound (II) is 46.70 (7)°.

## Supra­molecular features   

**Figure 3:**
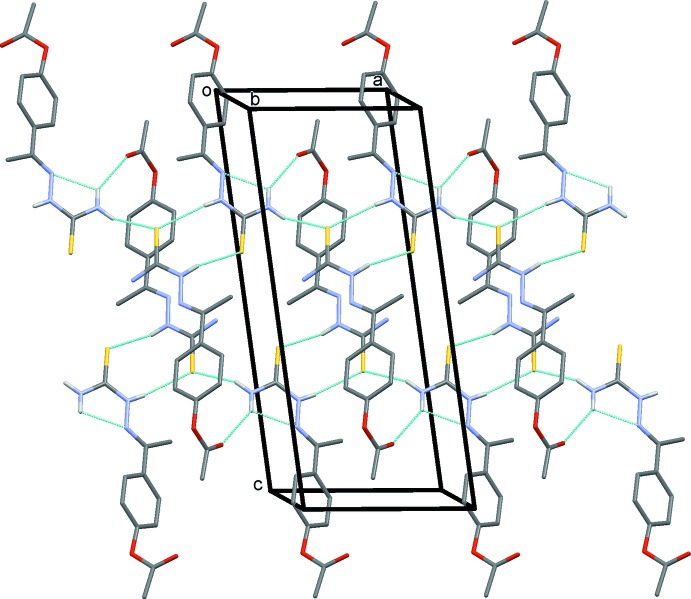
A view along the *b* axis of the crystal packing of compound (I). Hydrogen bonds are shown as dashed lines (see Table 1 ▸) and H atoms not involved in hydrogen bonds have been excluded for clarity.

In the crystal of (I), the two mol­ecules are linked by a pair of N—H⋯S hydrogen bonds forming *A*–*B* dimers with an 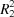
(8) ring motif. The dimers are linked by N—H⋯S and N—H⋯O hydrogen bonds, forming slabs lying parallel to (01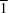), as shown in Table 1 ▸ and Fig. 3 ▸. Within the slabs there are N—H⋯π and C—H⋯π inter­actions present (Table 1 ▸).

**Figure 4:**
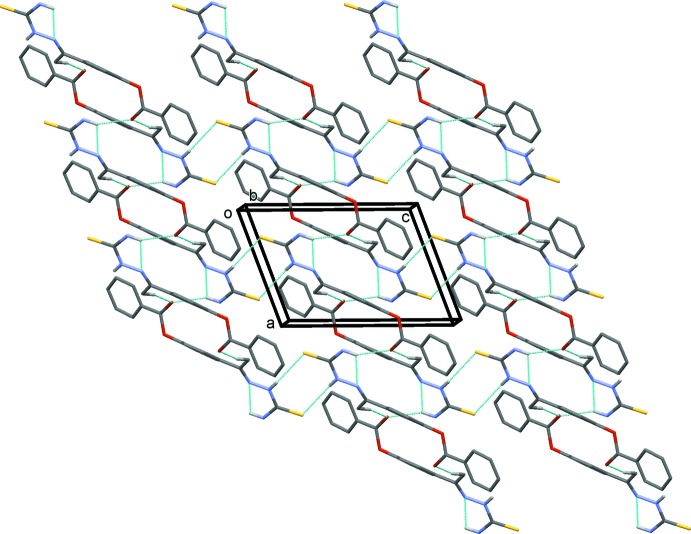
A view along the *b* axis of the crystal packing of compound (II). Hydrogen bonds are shown as dashed lines (see Table 2 ▸) and H atoms not involved in hydrogen bonds have been excluded for clarity.

In the crystal of (II), mol­ecules are linked by pairs of N—H⋯S hydrogen bonds, forming inversion dimers with an 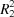
(8) ring motif (Table 2 ▸ and Fig. 4 ▸). As in the crystal of compound (I), the dimers are linked by N—H⋯S and N—H⋯O hydrogen bonds, forming slabs lying parallel to plane (01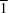); see Table 2 ▸ and Fig. 4 ▸. Within the slabs, there are only N—H⋯π inter­actions present (Table 2 ▸).

## Database survey   

A search of the Cambridge Structural Database (CSD, Version 5.37, last update May 2016; Groom *et al.*, 2016 ▸) for the substructure 2-(1-phenyl­ethyl­idene)hydrazine-1-carbo­thio­amide yielded 100 hits. One of the compounds, (*E*)-4-(*N*-carbamo­thioyl­ethane­hydrazono­yl)phenyl 4-methyl­benzoate (NOVFOV; Mani *et al.*, 2015 ▸) is the 4-methyl­benzoate analogue of compound (II). Like compound (I), it crystallizes with two independent mol­ecules in the asymmetric unit. The two mol­ecules differ essentially in the orientation of the hydrazinecarbo­thio­amide unit with respect to the central benzene ring. This dihedral angle is 5.95 (8)° in the first mol­ecule and 42.56 (9)° in the second. The benzoate groups are relatively planar and are inclined to the central benzene ring by 72.23 (7) and 53.10 (9)°, respectively, in the first and second mol­ecules. Hence, the conformation of the second mol­ecule resembles that of compound (II).

## Synthesis and crystallization   

**Compounds (I) and (II)**: Thio­semicarbazide (0.91g, 0.01 mol) was added to 50 ml of an ethano­lic solution of the 4-acetyl phenyl acetate (0.01 mol) for (I), and to an ethano­lic solution of the 4-acetyl­phenyl benzoate (0.01 mol) for (II), with continuous stirring for 4–5 h. The resulting mixtures were refluxed at 333 K and the purity of the products as well as composition of the reaction mixtures was monitored by TLC using ethyl acetate: hexane (3:7). The reaction mixtures were cooled to room temperature and the separated products were filtered, dried and finally recrystallized from chloro­form, solution, yielding block-like yellow crystals of (I) and pale-yellow crystals of (II).

## Refinement   

Crystal data, data collection and structure refinement details are summarized in Table 3 ▸. Hydrogen atoms were placed in calculated positions and refined as riding atoms: C—H = 0.93–0.96 Å and N—H = 0.86 Å, with *U*_iso_(H) = 1.5*U*_eq_(C-meth­yl) and 1.2*U*_eq_(C,N) for other H atoms.

## Supplementary Material

Crystal structure: contains datablock(s) global, I, II. DOI: 10.1107/S2056989016018983/su5323sup1.cif

Structure factors: contains datablock(s) I. DOI: 10.1107/S2056989016018983/su5323Isup2.hkl

Structure factors: contains datablock(s) II. DOI: 10.1107/S2056989016018983/su5323IIsup3.hkl

CCDC references: 1519492, 1519491

Additional supporting information:  crystallographic information; 3D view; checkCIF report

## Figures and Tables

**Figure 2:**
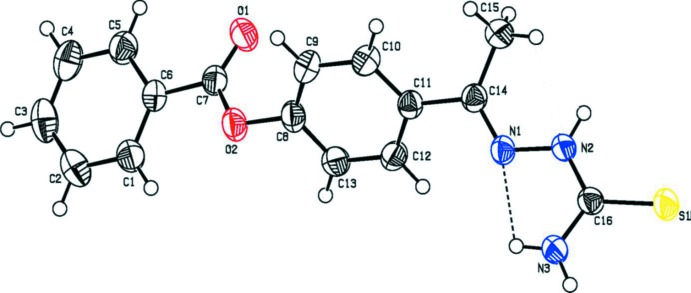
The mol­ecular structure of the compound (II), showing the atom labelling and displacement ellipsoids drawn at the 40% probability level. The short intra­molecular N—H⋯N contact is shown as a dashed line (see Table 2 ▸).

**Table 1:** Hydrogen-bond geometry (Å, °) for (I) *Cg*1 and *Cg*2 are the centroids of the C3*A*–C8*A* and C3*B*–C8*B* rings, respectively.

*D*—H⋯*A*	*D*—H	H⋯*A*	*D*⋯*A*	*D*—H⋯*A*
N3*A*—H3*A*1⋯N1*A*	0.86	2.26	2.617 (2)	105
N3*B*—H3*B*1⋯N1*B*	0.86	2.28	2.633 (2)	105
N2*A*—H2*A*⋯S1*B*	0.86	2.63	3.4724 (15)	167
N2*B*—H2*B*⋯S1*A*	0.86	2.71	3.4228 (16)	141
N3*A*—H3*A*1⋯O1*B* ^i^	0.86	2.44	3.164 (2)	142
N3*B*—H3*B*2⋯S1*A* ^ii^	0.86	2.57	3.4262 (17)	176
N3*A*—H3*A*2⋯*Cg*2^iii^	0.86	2.62	3.4763 (19)	130
C1*B*—H1*B*3⋯*Cg*1^iv^	0.96	2.73	3.691 (3)	154

Symmetry codes: (i) 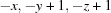; (ii) 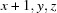; (iii) 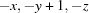; (iv) 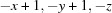.

**Table 2:** Hydrogen-bond geometry (Å, °) for (II) *Cg*2 is the centroid of the C8–C13 ring.

*D*—H⋯*A*	*D*—H	H⋯*A*	*D*⋯*A*	*D*—H⋯*A*
N3—H3*A*⋯N1	0.86	2.24	2.5953 (18)	105
N2—H2*A*⋯S1^i^	0.86	2.68	3.4697 (12)	153
N3—H3*A*⋯O1^ii^	0.86	2.27	3.0653 (15)	153
C15—H15*B*⋯O1^iii^	0.96	2.55	3.454 (2)	156
N3—H3*B*⋯*Cg*2^ii^	0.86	2.47	3.3385 (15)	122

Symmetry codes: (i) 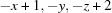; (ii) 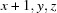; (iii) 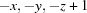.

**Table 3:** Experimental details

	(I)	(II)
Crystal data		
Chemical formula	C_11_H_13_N_3_O_2_S	C_16_H_15_N_3_O_2_S
*M*_r_	251.30	313.37
Crystal system, space group	Triclinic, *P* 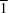	Triclinic, *P* 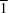
Temperature (K)	293	293
*a*, *b*, *c* (Å)	7.8783 (2), 8.9254 (3), 18.7372 (5)	7.8145 (4), 9.7538 (5), 10.9050 (7)
α, β, γ (°)	77.243 (2), 82.423 (2), 78.856 (2)	78.855 (4), 69.031 (2), 84.200 (3)
*V* (Å^3^)	1255.30 (6)	761.05 (8)
*Z*	4	2
Radiation type	Mo *K*α	Mo *K*α
μ (mm^−1^)	0.25	0.22
Crystal size (mm)	0.20 × 0.15 × 0.10	0.25 × 0.18 × 0.14

Data collection		
Diffractometer	Bruker SMART APEXII area-detector	Bruker SMART APEXII area-detector
Absorption correction	Multi-scan (*SADABS*; Bruker, 2008 ▸)	Multi-scan (*SADABS*; Bruker, 2008 ▸)
*T*_min_, *T*_max_	0.785, 0.854	0.745, 0.865
No. of measured, independent and observed [*I* > 2σ(*I*)] reflections	18970, 5128, 4104	11596, 3154, 2857
*R*_int_	0.023	0.027
(sin θ/λ)_max_ (Å^−1^)	0.625	0.628

Refinement		
*R*[*F* ^2^ > 2σ(*F* ^2^)], *wR*(*F* ^2^), *S*	0.038, 0.109, 1.03	0.034, 0.099, 1.05
No. of reflections	5128	3154
No. of parameters	311	201
H-atom treatment	H-atom parameters constrained	H-atom parameters constrained
Δρ_max_, Δρ_min_ (e Å^−3^)	0.25, −0.31	0.26, −0.29

Computer programs: *APEX2* and *SAINT* (Bruker, 2008 ▸), *SHELXS97* (Sheldrick, 2008 ▸), *SHELXL2014* (Sheldrick, 2015 ▸), *ORTEP-3 for Windows* (Farrugia, 2012 ▸), *Mercury* (Macrae *et al.*, 2008 ▸) and *PLATON* (Spek, 2009 ▸).

## supplementary crystallographic information

### (I) (E)-4-[1-(2-Carbamothioylhydrazinylidene)ethyl]phenyl acetate Crystal data

**Table:**

C_11_H_13_N_3_O_2_S	*Z* = 4
*M**_r_* = 251.30	*F*(000) = 528
Triclinic, *P*1	*D*_x_ = 1.330 Mg m^−^^3^
*a* = 7.8783 (2) Å	Mo *K*α radiation, λ = 0.71073 Å
*b* = 8.9254 (3) Å	Cell parameters from 5128 reflections
*c* = 18.7372 (5) Å	θ = 1.1–26.4°
α = 77.243 (2)°	µ = 0.25 mm^−^^1^
β = 82.423 (2)°	*T* = 293 K
γ = 78.856 (2)°	Block, yellow
*V* = 1255.30 (6) Å^3^	0.20 × 0.15 × 0.10 mm

### (I) (E)-4-[1-(2-Carbamothioylhydrazinylidene)ethyl]phenyl acetate Data collection

**Table:**

Bruker SMART APEXII area-detector diffractometer	4104 reflections with *I* > 2σ(*I*)
ω and φ scans	*R*_int_ = 0.023
Absorption correction: multi-scan (SADABS; Bruker, 2008)	θ_max_ = 26.4°, θ_min_ = 1.1°
*T*_min_ = 0.785, *T*_max_ = 0.854	*h* = −9→9
18970 measured reflections	*k* = −11→11
5128 independent reflections	*l* = −23→23

### (I) (E)-4-[1-(2-Carbamothioylhydrazinylidene)ethyl]phenyl acetate Refinement

**Table:**

Refinement on *F*^2^	Primary atom site location: structure-invariant direct methods
Least-squares matrix: full	Secondary atom site location: difference Fourier map
*R*[*F*^2^ > 2σ(*F*^2^)] = 0.038	Hydrogen site location: inferred from neighbouring sites
*wR*(*F*^2^) = 0.109	H-atom parameters constrained
*S* = 1.03	*w* = 1/[σ^2^(*F*_o_^2^) + (0.0498*P*)^2^ + 0.3822*P*] where *P* = (*F*_o_^2^ + 2*F*_c_^2^)/3
5128 reflections	(Δ/σ)_max_ = 0.001
311 parameters	Δρ_max_ = 0.25 e Å^−^^3^
0 restraints	Δρ_min_ = −0.31 e Å^−^^3^

### (I) (E)-4-[1-(2-Carbamothioylhydrazinylidene)ethyl]phenyl acetate Special details

**Table:**

Geometry. All esds (except the esd in the dihedral angle between two l.s. planes) are estimated using the full covariance matrix. The cell esds are taken into account individually in the estimation of esds in distances, angles and torsion angles; correlations between esds in cell parameters are only used when they are defined by crystal symmetry. An approximate (isotropic) treatment of cell esds is used for estimating esds involving l.s. planes.

### (I) (E)-4-[1-(2-Carbamothioylhydrazinylidene)ethyl]phenyl acetate Fractional atomic coordinates and isotropic or equivalent isotropic displacement parameters (Å^2^)

**Table:**

	*x*	*y*	*z*	*U*_iso_*/*U*_eq_	
C10B	0.2271 (3)	0.1728 (3)	0.52308 (11)	0.0616 (5)	
H10A	0.1335	0.2449	0.5001	0.092*	
H10B	0.2263	0.1858	0.5726	0.092*	
H10C	0.2129	0.0681	0.5235	0.092*	
C1A	0.0183 (3)	0.8835 (3)	1.22191 (11)	0.0706 (6)	
H1A1	0.0436	0.9807	1.2279	0.106*	
H1A2	0.0630	0.8015	1.2607	0.106*	
H1A3	−0.1051	0.8899	1.2236	0.106*	
C1B	0.5549 (3)	0.0841 (3)	0.07427 (10)	0.0632 (5)	
H1B1	0.4721	0.1283	0.0388	0.095*	
H1B2	0.5685	−0.0275	0.0835	0.095*	
H1B3	0.6646	0.1158	0.0556	0.095*	
C2A	0.1010 (3)	0.8500 (2)	1.15003 (10)	0.0506 (4)	
C2B	0.4920 (3)	0.1395 (2)	0.14379 (9)	0.0515 (4)	
C3A	0.0618 (2)	0.7263 (2)	1.05471 (9)	0.0438 (4)	
C3B	0.5256 (3)	0.0862 (2)	0.27010 (9)	0.0501 (4)	
C4A	−0.0202 (2)	0.7922 (2)	0.99205 (10)	0.0497 (4)	
H4A	−0.1086	0.8781	0.9916	0.060*	
C4B	0.6282 (3)	0.1679 (2)	0.29458 (10)	0.0564 (5)	
H4B	0.7253	0.1982	0.2652	0.068*	
C5A	0.0297 (2)	0.7298 (2)	0.92993 (9)	0.0476 (4)	
H5A	−0.0266	0.7738	0.8877	0.057*	
C5B	0.5867 (3)	0.2050 (2)	0.36322 (9)	0.0533 (5)	
H5B	0.6566	0.2603	0.3800	0.064*	
C6A	0.1629 (2)	0.60209 (19)	0.92929 (8)	0.0396 (4)	
C6B	0.4412 (2)	0.16043 (19)	0.40777 (9)	0.0438 (4)	
C7A	0.2445 (2)	0.5402 (2)	0.99344 (9)	0.0459 (4)	
H7A	0.3351	0.4559	0.9942	0.055*	
C7B	0.3427 (3)	0.0750 (2)	0.38147 (10)	0.0548 (5)	
H7B	0.2464	0.0423	0.4106	0.066*	
C8A	0.1934 (2)	0.6015 (2)	1.05617 (9)	0.0481 (4)	
H8A	0.2482	0.5581	1.0988	0.058*	
C8B	0.3850 (3)	0.0372 (2)	0.31264 (10)	0.0588 (5)	
H8B	0.3182	−0.0207	0.2958	0.071*	
C9A	0.2116 (2)	0.5324 (2)	0.86296 (8)	0.0412 (4)	
C9B	0.3961 (2)	0.20310 (19)	0.48111 (9)	0.0427 (4)	
C10A	0.3766 (3)	0.4199 (3)	0.85640 (11)	0.0705 (6)	
H10D	0.4646	0.4736	0.8273	0.106*	
H10E	0.3581	0.3395	0.8332	0.106*	
H10F	0.4132	0.3743	0.9045	0.106*	
C11A	0.0194 (2)	0.5408 (2)	0.70414 (9)	0.0428 (4)	
C11B	0.6013 (2)	0.3524 (2)	0.59815 (9)	0.0448 (4)	
N1A	0.10302 (18)	0.57512 (17)	0.81405 (7)	0.0423 (3)	
N1B	0.50947 (19)	0.26330 (17)	0.50292 (7)	0.0460 (3)	
N2A	0.14402 (18)	0.52010 (17)	0.74940 (7)	0.0444 (3)	
H2A	0.2473	0.4736	0.7384	0.053*	
N2B	0.47614 (19)	0.30636 (18)	0.57038 (7)	0.0483 (4)	
H2B	0.3756	0.3037	0.5945	0.058*	
N3A	−0.1399 (2)	0.5972 (2)	0.72772 (8)	0.0602 (4)	
H3A1	−0.1609	0.6193	0.7708	0.072*	
H3A2	−0.2229	0.6120	0.7001	0.072*	
N3B	0.7503 (2)	0.3608 (2)	0.55723 (9)	0.0641 (5)	
H3B1	0.7642	0.3371	0.5145	0.077*	
H3B2	0.8335	0.3898	0.5733	0.077*	
O1A	0.2381 (2)	0.8767 (2)	1.12093 (9)	0.0800 (5)	
O1B	0.3862 (2)	0.2510 (2)	0.15026 (8)	0.0863 (5)	
O2A	−0.00302 (17)	0.78178 (16)	1.11913 (7)	0.0555 (3)	
O2B	0.57246 (19)	0.04701 (15)	0.20057 (7)	0.0607 (4)	
S1A	0.07063 (6)	0.49355 (7)	0.62043 (2)	0.05713 (16)	
S1B	0.56754 (6)	0.39051 (7)	0.68325 (2)	0.05582 (15)	

### (I) (E)-4-[1-(2-Carbamothioylhydrazinylidene)ethyl]phenyl acetate Atomic displacement parameters (Å^2^)

**Table:**

	*U*^11^	*U*^22^	*U*^33^	*U*^12^	*U*^13^	*U*^23^
C10B	0.0553 (12)	0.0813 (14)	0.0553 (11)	−0.0165 (11)	0.0010 (9)	−0.0283 (10)
C1A	0.0818 (15)	0.0834 (15)	0.0541 (11)	−0.0020 (12)	−0.0163 (11)	−0.0344 (11)
C1B	0.0750 (14)	0.0799 (14)	0.0428 (10)	−0.0222 (11)	0.0034 (9)	−0.0265 (9)
C2A	0.0566 (12)	0.0518 (10)	0.0477 (10)	−0.0062 (9)	−0.0142 (9)	−0.0161 (8)
C2B	0.0558 (11)	0.0610 (11)	0.0406 (9)	−0.0078 (10)	−0.0053 (8)	−0.0179 (8)
C3A	0.0435 (9)	0.0564 (10)	0.0378 (8)	−0.0175 (8)	0.0009 (7)	−0.0177 (7)
C3B	0.0664 (12)	0.0445 (9)	0.0384 (9)	0.0034 (9)	−0.0108 (8)	−0.0140 (7)
C4A	0.0490 (10)	0.0543 (10)	0.0488 (10)	−0.0039 (8)	−0.0074 (8)	−0.0192 (8)
C4B	0.0699 (13)	0.0590 (11)	0.0427 (9)	−0.0163 (10)	0.0035 (9)	−0.0158 (8)
C5A	0.0514 (10)	0.0539 (10)	0.0396 (9)	−0.0061 (8)	−0.0110 (7)	−0.0126 (7)
C5B	0.0665 (12)	0.0565 (10)	0.0428 (9)	−0.0181 (9)	−0.0010 (8)	−0.0181 (8)
C6A	0.0379 (9)	0.0493 (9)	0.0352 (8)	−0.0144 (7)	−0.0021 (6)	−0.0106 (7)
C6B	0.0530 (10)	0.0417 (9)	0.0364 (8)	−0.0031 (8)	−0.0094 (7)	−0.0086 (7)
C7A	0.0440 (10)	0.0544 (10)	0.0394 (8)	−0.0043 (8)	−0.0064 (7)	−0.0118 (7)
C7B	0.0580 (12)	0.0644 (11)	0.0464 (10)	−0.0153 (9)	−0.0054 (8)	−0.0157 (8)
C8A	0.0500 (10)	0.0623 (11)	0.0340 (8)	−0.0116 (9)	−0.0081 (7)	−0.0095 (7)
C8B	0.0694 (13)	0.0628 (12)	0.0530 (11)	−0.0124 (10)	−0.0154 (10)	−0.0235 (9)
C9A	0.0368 (9)	0.0536 (10)	0.0352 (8)	−0.0112 (7)	−0.0013 (7)	−0.0113 (7)
C9B	0.0452 (9)	0.0456 (9)	0.0365 (8)	−0.0022 (7)	−0.0078 (7)	−0.0093 (7)
C10A	0.0578 (13)	0.1042 (17)	0.0488 (11)	0.0159 (12)	−0.0143 (9)	−0.0341 (11)
C11A	0.0387 (9)	0.0554 (10)	0.0362 (8)	−0.0082 (8)	−0.0039 (7)	−0.0130 (7)
C11B	0.0403 (9)	0.0559 (10)	0.0388 (8)	−0.0021 (8)	−0.0052 (7)	−0.0153 (7)
N1A	0.0410 (8)	0.0560 (8)	0.0335 (7)	−0.0104 (6)	−0.0013 (6)	−0.0158 (6)
N1B	0.0477 (8)	0.0579 (9)	0.0338 (7)	−0.0041 (7)	−0.0047 (6)	−0.0156 (6)
N2A	0.0355 (7)	0.0657 (9)	0.0349 (7)	−0.0049 (7)	−0.0024 (6)	−0.0202 (6)
N2B	0.0397 (8)	0.0720 (10)	0.0371 (7)	−0.0072 (7)	−0.0011 (6)	−0.0228 (7)
N3A	0.0410 (9)	0.0971 (13)	0.0450 (8)	0.0060 (8)	−0.0084 (7)	−0.0322 (8)
N3B	0.0451 (9)	0.1090 (14)	0.0489 (9)	−0.0192 (9)	0.0046 (7)	−0.0384 (9)
O1A	0.0727 (11)	0.1024 (12)	0.0834 (11)	−0.0401 (10)	0.0012 (8)	−0.0408 (9)
O1B	0.1032 (13)	0.0907 (11)	0.0507 (8)	0.0349 (10)	−0.0177 (8)	−0.0223 (8)
O2A	0.0525 (8)	0.0791 (9)	0.0448 (7)	−0.0190 (7)	0.0029 (6)	−0.0311 (6)
O2B	0.0776 (9)	0.0599 (8)	0.0442 (7)	0.0098 (7)	−0.0114 (6)	−0.0246 (6)
S1A	0.0422 (3)	0.0971 (4)	0.0381 (2)	−0.0090 (2)	−0.00201 (18)	−0.0298 (2)
S1B	0.0444 (3)	0.0874 (4)	0.0418 (2)	−0.0056 (2)	−0.00359 (19)	−0.0310 (2)

### (I) (E)-4-[1-(2-Carbamothioylhydrazinylidene)ethyl]phenyl acetate Geometric parameters (Å, º)

**Table:**

C10B—C9B	1.494 (3)	C6A—C7A	1.392 (2)
C10B—H10A	0.9600	C6A—C9A	1.484 (2)
C10B—H10B	0.9600	C6B—C7B	1.387 (2)
C10B—H10C	0.9600	C6B—C9B	1.487 (2)
C1A—C2A	1.483 (3)	C7A—C8A	1.385 (2)
C1A—H1A1	0.9600	C7A—H7A	0.9300
C1A—H1A2	0.9600	C7B—C8B	1.387 (2)
C1A—H1A3	0.9600	C7B—H7B	0.9300
C1B—C2B	1.485 (2)	C8A—H8A	0.9300
C1B—H1B1	0.9600	C8B—H8B	0.9300
C1B—H1B2	0.9600	C9A—N1A	1.282 (2)
C1B—H1B3	0.9600	C9A—C10A	1.490 (3)
C2A—O1A	1.186 (2)	C9B—N1B	1.278 (2)
C2A—O2A	1.361 (2)	C10A—H10D	0.9600
C2B—O1B	1.186 (2)	C10A—H10E	0.9600
C2B—O2B	1.343 (2)	C10A—H10F	0.9600
C3A—C8A	1.366 (3)	C11A—N3A	1.315 (2)
C3A—C4A	1.375 (2)	C11A—N2A	1.341 (2)
C3A—O2A	1.4023 (19)	C11A—S1A	1.6915 (16)
C3B—C8B	1.361 (3)	C11B—N3B	1.320 (2)
C3B—C4B	1.367 (3)	C11B—N2B	1.341 (2)
C3B—O2B	1.4074 (19)	C11B—S1B	1.6799 (16)
C4A—C5A	1.379 (2)	N1A—N2A	1.3825 (17)
C4A—H4A	0.9300	N1B—N2B	1.3792 (18)
C4B—C5B	1.381 (2)	N2A—H2A	0.8600
C4B—H4B	0.9300	N2B—H2B	0.8600
C5A—C6A	1.394 (2)	N3A—H3A1	0.8600
C5A—H5A	0.9300	N3A—H3A2	0.8600
C5B—C6B	1.395 (3)	N3B—H3B1	0.8600
C5B—H5B	0.9300	N3B—H3B2	0.8600
			
C9B—C10B—H10A	109.5	C5B—C6B—C9B	120.40 (15)
C9B—C10B—H10B	109.5	C8A—C7A—C6A	121.27 (17)
H10A—C10B—H10B	109.5	C8A—C7A—H7A	119.4
C9B—C10B—H10C	109.5	C6A—C7A—H7A	119.4
H10A—C10B—H10C	109.5	C8B—C7B—C6B	121.33 (18)
H10B—C10B—H10C	109.5	C8B—C7B—H7B	119.3
C2A—C1A—H1A1	109.5	C6B—C7B—H7B	119.3
C2A—C1A—H1A2	109.5	C3A—C8A—C7A	119.29 (16)
H1A1—C1A—H1A2	109.5	C3A—C8A—H8A	120.4
C2A—C1A—H1A3	109.5	C7A—C8A—H8A	120.4
H1A1—C1A—H1A3	109.5	C3B—C8B—C7B	119.06 (17)
H1A2—C1A—H1A3	109.5	C3B—C8B—H8B	120.5
C2B—C1B—H1B1	109.5	C7B—C8B—H8B	120.5
C2B—C1B—H1B2	109.5	N1A—C9A—C6A	115.51 (15)
H1B1—C1B—H1B2	109.5	N1A—C9A—C10A	124.10 (15)
C2B—C1B—H1B3	109.5	C6A—C9A—C10A	120.39 (14)
H1B1—C1B—H1B3	109.5	N1B—C9B—C6B	115.48 (15)
H1B2—C1B—H1B3	109.5	N1B—C9B—C10B	125.51 (15)
O1A—C2A—O2A	122.19 (17)	C6B—C9B—C10B	119.01 (15)
O1A—C2A—C1A	127.08 (18)	C9A—C10A—H10D	109.5
O2A—C2A—C1A	110.72 (17)	C9A—C10A—H10E	109.5
O1B—C2B—O2B	122.74 (16)	H10D—C10A—H10E	109.5
O1B—C2B—C1B	126.04 (18)	C9A—C10A—H10F	109.5
O2B—C2B—C1B	111.22 (17)	H10D—C10A—H10F	109.5
C8A—C3A—C4A	121.15 (15)	H10E—C10A—H10F	109.5
C8A—C3A—O2A	120.42 (15)	N3A—C11A—N2A	117.60 (14)
C4A—C3A—O2A	118.20 (16)	N3A—C11A—S1A	122.72 (13)
C8B—C3B—C4B	121.45 (16)	N2A—C11A—S1A	119.68 (12)
C8B—C3B—O2B	119.99 (17)	N3B—C11B—N2B	117.47 (15)
C4B—C3B—O2B	118.50 (17)	N3B—C11B—S1B	122.55 (13)
C3A—C4A—C5A	119.40 (17)	N2B—C11B—S1B	119.94 (13)
C3A—C4A—H4A	120.3	C9A—N1A—N2A	118.53 (14)
C5A—C4A—H4A	120.3	C9B—N1B—N2B	118.72 (14)
C3B—C4B—C5B	119.52 (18)	C11A—N2A—N1A	118.60 (13)
C3B—C4B—H4B	120.2	C11A—N2A—H2A	120.7
C5B—C4B—H4B	120.2	N1A—N2A—H2A	120.7
C4A—C5A—C6A	121.19 (16)	C11B—N2B—N1B	119.63 (14)
C4A—C5A—H5A	119.4	C11B—N2B—H2B	120.2
C6A—C5A—H5A	119.4	N1B—N2B—H2B	120.2
C4B—C5B—C6B	120.83 (17)	C11A—N3A—H3A1	120.0
C4B—C5B—H5B	119.6	C11A—N3A—H3A2	120.0
C6B—C5B—H5B	119.6	H3A1—N3A—H3A2	120.0
C7A—C6A—C5A	117.68 (15)	C11B—N3B—H3B1	120.0
C7A—C6A—C9A	121.51 (15)	C11B—N3B—H3B2	120.0
C5A—C6A—C9A	120.79 (14)	H3B1—N3B—H3B2	120.0
C7B—C6B—C5B	117.77 (16)	C2A—O2A—C3A	118.74 (14)
C7B—C6B—C9B	121.82 (16)	C2B—O2B—C3B	117.30 (14)
			
C8A—C3A—C4A—C5A	0.9 (3)	C5A—C6A—C9A—C10A	166.64 (18)
O2A—C3A—C4A—C5A	−173.63 (15)	C7B—C6B—C9B—N1B	170.96 (17)
C8B—C3B—C4B—C5B	−1.6 (3)	C5B—C6B—C9B—N1B	−8.5 (2)
O2B—C3B—C4B—C5B	−178.73 (17)	C7B—C6B—C9B—C10B	−8.7 (3)
C3A—C4A—C5A—C6A	−0.6 (3)	C5B—C6B—C9B—C10B	171.80 (17)
C3B—C4B—C5B—C6B	−0.1 (3)	C6A—C9A—N1A—N2A	177.13 (13)
C4A—C5A—C6A—C7A	−0.3 (3)	C10A—C9A—N1A—N2A	−3.3 (3)
C4A—C5A—C6A—C9A	177.89 (16)	C6B—C9B—N1B—N2B	179.76 (14)
C4B—C5B—C6B—C7B	1.4 (3)	C10B—C9B—N1B—N2B	−0.6 (3)
C4B—C5B—C6B—C9B	−179.04 (17)	N3A—C11A—N2A—N1A	−7.3 (2)
C5A—C6A—C7A—C8A	1.0 (2)	S1A—C11A—N2A—N1A	173.06 (12)
C9A—C6A—C7A—C8A	−177.17 (15)	C9A—N1A—N2A—C11A	168.09 (16)
C5B—C6B—C7B—C8B	−1.2 (3)	N3B—C11B—N2B—N1B	3.0 (3)
C9B—C6B—C7B—C8B	179.30 (17)	S1B—C11B—N2B—N1B	−174.85 (12)
C4A—C3A—C8A—C7A	−0.2 (3)	C9B—N1B—N2B—C11B	172.12 (16)
O2A—C3A—C8A—C7A	174.20 (15)	O1A—C2A—O2A—C3A	7.0 (3)
C6A—C7A—C8A—C3A	−0.8 (3)	C1A—C2A—O2A—C3A	−173.17 (16)
C4B—C3B—C8B—C7B	1.8 (3)	C8A—C3A—O2A—C2A	67.9 (2)
O2B—C3B—C8B—C7B	178.94 (17)	C4A—C3A—O2A—C2A	−117.52 (19)
C6B—C7B—C8B—C3B	−0.4 (3)	O1B—C2B—O2B—C3B	0.6 (3)
C7A—C6A—C9A—N1A	164.41 (16)	C1B—C2B—O2B—C3B	179.85 (16)
C5A—C6A—C9A—N1A	−13.8 (2)	C8B—C3B—O2B—C2B	84.9 (2)
C7A—C6A—C9A—C10A	−15.2 (3)	C4B—C3B—O2B—C2B	−97.9 (2)

### (I) (E)-4-[1-(2-Carbamothioylhydrazinylidene)ethyl]phenyl acetate Hydrogen-bond geometry (Å, º)

Cg1 and Cg2 are the centroids of the C3*A*–C8*A* and C3*B*–C8*B* rings, respectively.

**Table:**

*D*—H···*A*	*D*—H	H···*A*	*D*···*A*	*D*—H···*A*
N3*A*—H3*A*1···N1*A*	0.86	2.26	2.617 (2)	105
N3*B*—H3*B*1···N1*B*	0.86	2.28	2.633 (2)	105
N2*A*—H2*A*···S1*B*	0.86	2.63	3.4724 (15)	167
N2*B*—H2*B*···S1*A*	0.86	2.71	3.4228 (16)	141
N3*A*—H3*A*1···O1*B*^i^	0.86	2.44	3.164 (2)	142
N3*B*—H3*B*2···S1*A*^ii^	0.86	2.57	3.4262 (17)	176
N3*A*—H3*A*2···*Cg*2^iii^	0.86	2.62	3.4763 (19)	130
C1*B*—H1*B*3···*Cg*1^iv^	0.96	2.73	3.691 (3)	154

Symmetry codes: (i) −*x*, −*y*+1, −*z*+1; (ii) *x*+1, *y*, *z*; (iii) −*x*, −*y*+1, −*z*; (iv) −*x*+1, −*y*+1, −*z*.

### (II) (E)-4-[1-(2-Carbamothioylhydrazinylidene)ethyl]phenyl benzoate Crystal data

**Table:**

C_16_H_15_N_3_O_2_S	*Z* = 2
*M**_r_* = 313.37	*F*(000) = 328
Triclinic, *P*1	*D*_x_ = 1.367 Mg m^−^^3^
*a* = 7.8145 (4) Å	Mo *K*α radiation, λ = 0.71073 Å
*b* = 9.7538 (5) Å	Cell parameters from 3154 reflections
*c* = 10.9050 (7) Å	θ = 2.0–26.5°
α = 78.855 (4)°	µ = 0.22 mm^−^^1^
β = 69.031 (2)°	*T* = 293 K
γ = 84.200 (3)°	Block, pale-yellow
*V* = 761.05 (8) Å^3^	0.25 × 0.18 × 0.14 mm

### (II) (E)-4-[1-(2-Carbamothioylhydrazinylidene)ethyl]phenyl benzoate Data collection

**Table:**

Bruker SMART APEXII area-detector diffractometer	2857 reflections with *I* > 2σ(*I*)
ω and φ scans	*R*_int_ = 0.027
Absorption correction: multi-scan (*SADABS*; Bruker, 2008)	θ_max_ = 26.5°, θ_min_ = 2.0°
*T*_min_ = 0.745, *T*_max_ = 0.865	*h* = −8→9
11596 measured reflections	*k* = −12→12
3154 independent reflections	*l* = −13→13

### (II) (E)-4-[1-(2-Carbamothioylhydrazinylidene)ethyl]phenyl benzoate Refinement

**Table:**

Refinement on *F*^2^	Hydrogen site location: inferred from neighbouring sites
Least-squares matrix: full	H-atom parameters constrained
*R*[*F*^2^ > 2σ(*F*^2^)] = 0.034	*w* = 1/[σ^2^(*F*_o_^2^) + (0.0512*P*)^2^ + 0.1914*P*] where *P* = (*F*_o_^2^ + 2*F*_c_^2^)/3
*wR*(*F*^2^) = 0.099	(Δ/σ)_max_ = 0.001
*S* = 1.05	Δρ_max_ = 0.26 e Å^−^^3^
3154 reflections	Δρ_min_ = −0.29 e Å^−^^3^
201 parameters	Extinction correction: SHELXL2014 (Sheldrick, 2015), Fc^*^=kFc[1+0.001xFc^2^λ^3^/sin(2θ)]^-1/4^
0 restraints	Extinction coefficient: 0.080 (6)

### (II) (E)-4-[1-(2-Carbamothioylhydrazinylidene)ethyl]phenyl benzoate Special details

**Table:**

Geometry. All esds (except the esd in the dihedral angle between two l.s. planes) are estimated using the full covariance matrix. The cell esds are taken into account individually in the estimation of esds in distances, angles and torsion angles; correlations between esds in cell parameters are only used when they are defined by crystal symmetry. An approximate (isotropic) treatment of cell esds is used for estimating esds involving l.s. planes.

### (II) (E)-4-[1-(2-Carbamothioylhydrazinylidene)ethyl]phenyl benzoate Fractional atomic coordinates and isotropic or equivalent isotropic displacement parameters (Å^2^)

**Table:**

	*x*	*y*	*z*	*U*_iso_*/*U*_eq_	
C1	−0.0574 (2)	0.60395 (15)	0.12357 (14)	0.0461 (3)	
H1	0.0581	0.6165	0.1252	0.055*	
C2	−0.1286 (2)	0.69706 (17)	0.03984 (16)	0.0562 (4)	
H2	−0.0616	0.7736	−0.0138	0.067*	
C3	−0.2978 (3)	0.67730 (18)	0.03519 (16)	0.0596 (4)	
H3	−0.3439	0.7398	−0.0223	0.072*	
C4	−0.3993 (2)	0.56519 (19)	0.11541 (17)	0.0579 (4)	
H4	−0.5134	0.5519	0.1117	0.069*	
C5	−0.3315 (2)	0.47264 (16)	0.20141 (15)	0.0483 (3)	
H5	−0.4005	0.3977	0.2567	0.058*	
C6	−0.15975 (18)	0.49176 (14)	0.20510 (12)	0.0384 (3)	
C7	−0.09783 (18)	0.39381 (14)	0.30353 (12)	0.0392 (3)	
C8	0.15463 (18)	0.31751 (15)	0.37198 (13)	0.0400 (3)	
C9	0.15816 (19)	0.17391 (15)	0.39018 (14)	0.0442 (3)	
H9	0.1159	0.1286	0.3395	0.053*	
C10	0.22566 (19)	0.09777 (14)	0.48531 (14)	0.0412 (3)	
H10	0.2247	0.0006	0.5004	0.049*	
C11	0.29489 (16)	0.16457 (13)	0.55862 (12)	0.0346 (3)	
C12	0.29666 (19)	0.31025 (14)	0.53336 (14)	0.0406 (3)	
H12	0.3466	0.3565	0.5790	0.049*	
C13	0.22498 (19)	0.38662 (14)	0.44118 (14)	0.0433 (3)	
H13	0.2243	0.4838	0.4260	0.052*	
C14	0.36348 (17)	0.08555 (13)	0.66391 (12)	0.0353 (3)	
C15	0.2849 (2)	−0.05203 (16)	0.73732 (16)	0.0549 (4)	
H15A	0.3642	−0.1019	0.7820	0.082*	
H15B	0.2737	−0.1056	0.6755	0.082*	
H15C	0.1661	−0.0373	0.8018	0.082*	
C16	0.70223 (17)	0.13075 (13)	0.78932 (12)	0.0358 (3)	
N1	0.48733 (14)	0.14652 (11)	0.68311 (10)	0.0361 (2)	
N2	0.54777 (15)	0.08471 (11)	0.78611 (11)	0.0374 (2)	
H2A	0.4883	0.0188	0.8464	0.045*	
N3	0.79854 (16)	0.21798 (13)	0.68277 (11)	0.0467 (3)	
H3A	0.7611	0.2423	0.6162	0.056*	
H3B	0.8984	0.2502	0.6803	0.056*	
O1	−0.19599 (15)	0.31803 (12)	0.39711 (10)	0.0550 (3)	
O2	0.08423 (13)	0.40017 (11)	0.27880 (9)	0.0477 (3)	
S1	0.77088 (5)	0.07598 (4)	0.92056 (4)	0.05405 (15)	

### (II) (E)-4-[1-(2-Carbamothioylhydrazinylidene)ethyl]phenyl benzoate Atomic displacement parameters (Å^2^)

**Table:**

	*U*^11^	*U*^22^	*U*^33^	*U*^12^	*U*^13^	*U*^23^
C1	0.0487 (8)	0.0491 (8)	0.0399 (7)	−0.0026 (6)	−0.0165 (6)	−0.0032 (6)
C2	0.0671 (10)	0.0487 (8)	0.0458 (8)	0.0029 (7)	−0.0183 (7)	0.0033 (6)
C3	0.0728 (11)	0.0613 (10)	0.0465 (8)	0.0207 (8)	−0.0300 (8)	−0.0070 (7)
C4	0.0528 (9)	0.0721 (10)	0.0583 (9)	0.0112 (8)	−0.0323 (8)	−0.0149 (8)
C5	0.0451 (8)	0.0555 (8)	0.0467 (8)	−0.0013 (6)	−0.0206 (6)	−0.0055 (6)
C6	0.0423 (7)	0.0432 (7)	0.0315 (6)	0.0014 (5)	−0.0157 (5)	−0.0067 (5)
C7	0.0409 (7)	0.0454 (7)	0.0333 (6)	−0.0030 (5)	−0.0159 (5)	−0.0048 (5)
C8	0.0341 (6)	0.0523 (7)	0.0320 (6)	−0.0008 (5)	−0.0134 (5)	0.0004 (5)
C9	0.0457 (7)	0.0526 (8)	0.0419 (7)	0.0009 (6)	−0.0224 (6)	−0.0131 (6)
C10	0.0426 (7)	0.0409 (7)	0.0447 (7)	0.0023 (5)	−0.0196 (6)	−0.0112 (5)
C11	0.0308 (6)	0.0398 (6)	0.0328 (6)	0.0008 (5)	−0.0115 (5)	−0.0055 (5)
C12	0.0425 (7)	0.0419 (7)	0.0429 (7)	−0.0020 (5)	−0.0211 (6)	−0.0073 (5)
C13	0.0451 (7)	0.0394 (7)	0.0473 (7)	−0.0026 (5)	−0.0214 (6)	−0.0006 (5)
C14	0.0332 (6)	0.0384 (6)	0.0345 (6)	0.0000 (5)	−0.0128 (5)	−0.0054 (5)
C15	0.0616 (10)	0.0520 (8)	0.0568 (9)	−0.0181 (7)	−0.0328 (8)	0.0094 (7)
C16	0.0333 (6)	0.0364 (6)	0.0365 (6)	−0.0022 (5)	−0.0130 (5)	−0.0010 (5)
N1	0.0360 (5)	0.0396 (5)	0.0339 (5)	−0.0005 (4)	−0.0162 (4)	−0.0013 (4)
N2	0.0365 (6)	0.0404 (6)	0.0354 (5)	−0.0073 (4)	−0.0166 (4)	0.0047 (4)
N3	0.0427 (6)	0.0574 (7)	0.0394 (6)	−0.0168 (5)	−0.0187 (5)	0.0098 (5)
O1	0.0480 (6)	0.0675 (7)	0.0456 (6)	−0.0137 (5)	−0.0208 (5)	0.0146 (5)
O2	0.0404 (5)	0.0622 (6)	0.0387 (5)	−0.0055 (4)	−0.0193 (4)	0.0083 (4)
S1	0.0454 (2)	0.0720 (3)	0.0464 (2)	−0.01842 (18)	−0.02749 (17)	0.01713 (18)

### (II) (E)-4-[1-(2-Carbamothioylhydrazinylidene)ethyl]phenyl benzoate Geometric parameters (Å, º)

**Table:**

C1—C2	1.381 (2)	C10—C11	1.3923 (18)
C1—C6	1.3853 (19)	C10—H10	0.9300
C1—H1	0.9300	C11—C12	1.3947 (18)
C2—C3	1.375 (3)	C11—C14	1.4881 (17)
C2—H2	0.9300	C12—C13	1.3815 (18)
C3—C4	1.378 (3)	C12—H12	0.9300
C3—H3	0.9300	C13—H13	0.9300
C4—C5	1.381 (2)	C14—N1	1.2820 (16)
C4—H4	0.9300	C14—C15	1.4894 (18)
C5—C6	1.389 (2)	C15—H15A	0.9600
C5—H5	0.9300	C15—H15B	0.9600
C6—C7	1.4767 (17)	C15—H15C	0.9600
C7—O1	1.1995 (16)	C16—N3	1.3273 (16)
C7—O2	1.3555 (16)	C16—N2	1.3437 (16)
C8—C9	1.375 (2)	C16—S1	1.6785 (13)
C8—C13	1.376 (2)	N1—N2	1.3816 (14)
C8—O2	1.4067 (15)	N2—H2A	0.8600
C9—C10	1.3858 (19)	N3—H3A	0.8600
C9—H9	0.9300	N3—H3B	0.8600
			
C2—C1—C6	119.40 (14)	C10—C11—C12	118.52 (12)
C2—C1—H1	120.3	C10—C11—C14	122.07 (11)
C6—C1—H1	120.3	C12—C11—C14	119.40 (11)
C3—C2—C1	120.47 (15)	C13—C12—C11	120.66 (12)
C3—C2—H2	119.8	C13—C12—H12	119.7
C1—C2—H2	119.8	C11—C12—H12	119.7
C2—C3—C4	120.27 (14)	C8—C13—C12	119.37 (13)
C2—C3—H3	119.9	C8—C13—H13	120.3
C4—C3—H3	119.9	C12—C13—H13	120.3
C3—C4—C5	119.92 (16)	N1—C14—C11	114.67 (11)
C3—C4—H4	120.0	N1—C14—C15	126.07 (12)
C5—C4—H4	120.0	C11—C14—C15	119.26 (11)
C4—C5—C6	119.80 (14)	C14—C15—H15A	109.5
C4—C5—H5	120.1	C14—C15—H15B	109.5
C6—C5—H5	120.1	H15A—C15—H15B	109.5
C1—C6—C5	120.12 (13)	C14—C15—H15C	109.5
C1—C6—C7	122.27 (12)	H15A—C15—H15C	109.5
C5—C6—C7	117.51 (12)	H15B—C15—H15C	109.5
O1—C7—O2	122.72 (12)	N3—C16—N2	116.66 (11)
O1—C7—C6	124.71 (12)	N3—C16—S1	122.30 (10)
O2—C7—C6	112.56 (11)	N2—C16—S1	121.02 (9)
C9—C8—C13	121.44 (12)	C14—N1—N2	118.03 (10)
C9—C8—O2	121.45 (12)	C16—N2—N1	117.98 (10)
C13—C8—O2	117.08 (12)	C16—N2—H2A	121.0
C8—C9—C10	118.95 (12)	N1—N2—H2A	121.0
C8—C9—H9	120.5	C16—N3—H3A	120.0
C10—C9—H9	120.5	C16—N3—H3B	120.0
C9—C10—C11	120.96 (12)	H3A—N3—H3B	120.0
C9—C10—H10	119.5	C7—O2—C8	116.96 (10)
C11—C10—H10	119.5		
			
C6—C1—C2—C3	−1.3 (2)	C14—C11—C12—C13	−176.57 (12)
C1—C2—C3—C4	0.8 (3)	C9—C8—C13—C12	−1.6 (2)
C2—C3—C4—C5	0.4 (3)	O2—C8—C13—C12	−179.67 (12)
C3—C4—C5—C6	−1.0 (2)	C11—C12—C13—C8	−1.3 (2)
C2—C1—C6—C5	0.7 (2)	C10—C11—C14—N1	152.10 (12)
C2—C1—C6—C7	−175.72 (13)	C12—C11—C14—N1	−28.98 (17)
C4—C5—C6—C1	0.5 (2)	C10—C11—C14—C15	−28.60 (19)
C4—C5—C6—C7	177.06 (13)	C12—C11—C14—C15	150.32 (14)
C1—C6—C7—O1	160.66 (14)	C11—C14—N1—N2	175.16 (10)
C5—C6—C7—O1	−15.8 (2)	C15—C14—N1—N2	−4.1 (2)
C1—C6—C7—O2	−18.01 (18)	N3—C16—N2—N1	−9.43 (18)
C5—C6—C7—O2	165.53 (12)	S1—C16—N2—N1	172.24 (9)
C13—C8—C9—C10	3.3 (2)	C14—N1—N2—C16	166.17 (12)
O2—C8—C9—C10	−178.70 (12)	O1—C7—O2—C8	−2.8 (2)
C8—C9—C10—C11	−2.2 (2)	C6—C7—O2—C8	175.91 (11)
C9—C10—C11—C12	−0.6 (2)	C9—C8—O2—C7	66.31 (17)
C9—C10—C11—C14	178.29 (12)	C13—C8—O2—C7	−115.64 (14)
C10—C11—C12—C13	2.4 (2)		

### (II) (E)-4-[1-(2-Carbamothioylhydrazinylidene)ethyl]phenyl benzoate Hydrogen-bond geometry (Å, º)

Cg2 is the centroid of the C8–C13 ring.

**Table:**

*D*—H···*A*	*D*—H	H···*A*	*D*···*A*	*D*—H···*A*
N3—H3*A*···N1	0.86	2.24	2.5953 (18)	105
N2—H2*A*···S1^i^	0.86	2.68	3.4697 (12)	153
N3—H3*A*···O1^ii^	0.86	2.27	3.0653 (15)	153
C15—H15*B*···O1^iii^	0.96	2.55	3.454 (2)	156
N3—H3*B*···*Cg*2^ii^	0.86	2.47	3.3385 (15)	122

Symmetry codes: (i) −*x*+1, −*y*, −*z*+2; (ii) *x*+1, *y*, *z*; (iii) −*x*, −*y*, −*z*+1.
